# Effectiveness of a Mobile Health Intervention (DOT Selfie) in Increasing Treatment Adherence Monitoring and Support for Patients With Tuberculosis in Uganda: Randomized Controlled Trial

**DOI:** 10.2196/57991

**Published:** 2025-01-16

**Authors:** Juliet Nabbuye Sekandi, Esther Buregyeya, Sarah Zalwango, Damalie Nakkonde, Patrick Kaggwa, Trang Ho Thu Quach, David Asiimwe, Lynn Atuyambe, Kevin Dobbin

**Affiliations:** 1 Department of Epidemiology and Biostatistics College of Public Health University of Georgia Athens, GA United States; 2 Global Health Institute College of Public Health University of Georgia Athens, GA United States; 3 School of Public Health College of Health Sciences Makerere University Kampala Uganda; 4 Department of Public Health Services and Environment Kampala Capital City Aunthority Kampala Uganda; 5 Uganda National Tuberculosis and Leprosy Control Programme Ministry of Health Kampala Uganda

**Keywords:** tuberculosis, digital adherence technologies, video-observed treatment, video directly observed treatment, directly observed therapy, adherence, mHealth, Uganda, Africa

## Abstract

**Background:**

Directly observed therapy (DOT) is the standard method for monitoring adherence to tuberculosis (TB) treatment. However, implementing DOT poses challenges for both patients and providers due to limited financial and human resources. Increasing evidence suggests that emerging digital adherence technologies, such as video directly observed therapy (VDOT), can serve as viable alternatives.

**Objective:**

This study aims to evaluate the effectiveness of VDOT compared with usual care directly observed therapy (UCDOT).

**Methods:**

Between July 2020 and October 2021, we conducted a 2-arm, parallel-group, open-label randomized trial with a 1:1 assignment to receive either the VDOT intervention (n=72) or UCDOT (n=72) for treatment adherence monitoring at public health clinics in Kampala, Uganda. Each group was further stratified to ensure equal numbers of males and females. Eligible patients were aged 18-65 years, had a confirmed diagnosis of TB, and were undergoing daily treatment. The VDOT group was provided with a smartphone equipped with an app, while the UCDOT group followed the routine monitoring practices outlined by the Uganda National TB Program. We tested the hypothesis that VDOT was more effective than UCDOT for monitoring medication adherence. The primary outcome was adherence, defined as having ≥80% of the expected doses observed during the 6-month treatment period. An intention-to-treat analysis was conducted, and multivariable logistic regression was used to estimate the effect of the intervention on adherence monitoring. Adjusted relative risk ratios and their corresponding 95% CIs are presented. Secondary outcomes included treatment completion, loss to follow-up, death, and reasons for missed videos in the intervention group.

**Results:**

The intention-to-treat analysis included 142 participants, with 2 excluded due to discontinuation of medication within the first week after enrollment. The median age of participants was 34 (IQR 26-45) years. The median fraction of expected doses observed (FEDO) was significantly higher in the VDOT group compared with the UCDOT group (100, IQR 80-100 vs 30, IQR 10-60, respectively; *P*<.001). When using a FEDO cutoff of ≥80% to define optimal adherence, 63 of 142 (44%) patients met the threshold, with a significant difference between the VDOT and UCDOT groups (56/71, 79% vs 7/71, 10%, *P*<.001). After adjusting for confounders, VDOT users were significantly more likely to achieve ≥80% of their expected doses observed compared with UCDOT users (adjusted risk ratio 8.4, 95% CI 4.16-17.0). The most common reasons for failing to submit videos of medication intake were an uncharged phone battery, forgetting to record videos during medication intake, and losing the smartphone.

**Conclusions:**

Enhanced VDOT was more effective than UCDOT in increasing adherence monitoring among patients with TB in Uganda. This evidence highlights the potential of digital technologies to improve treatment adherence monitoring and support in high TB burden settings with limited human resources.

**Trial Registration:**

ClinicalTrials.gov NCT04134689; http://clinicaltrials.gov/ct2/show/NCT04134689

## Introduction

The End TB Strategy envisions a world free of tuberculosis (TB), with zero deaths, disease, and suffering due to TB by 2035 [1]. In 2022, an estimated 10.6 million new cases were reported, while 1.6 million people died from TB worldwide [2]. Although effective treatments for TB disease have existed for over 50 years, nonadherence to medication remains a common problem among patients and poses a significant obstacle to achieving the goals of the End TB Strategy [1,3]. Nonadherence reduces cure rates, prolongs infectiousness, and results in poor treatment outcomes, including treatment failure, drug resistance, death, and relapse [4-7]. The emergence of drug-resistant TB and multidrug-resistant TB (MDR-TB) is partly attributed to patients not adhering to their medication properly [4]. Globally, an estimated 450,000 incident cases of MDR-TB were reported in 2021, marking a 3.1% increase from 437,000 cases in 2020 [2]. According to a recent meta-analysis that included 148 studies, the global pooled prevalence of MDR-TB was 11.6% (95% CI 9.1%-14.5%), significantly higher than previous estimates [8].

The World Health Organization (WHO) and US clinical practice guidelines recommend directly observed therapy (DOT) as the standard of care for TB treatment [9,10]. DOT involves a health care worker observing a patient in person while they ingest TB medications to monitor adherence and provide support as needed [10]. When implemented correctly, in-person DOT can facilitate high levels of medication adherence and successful treatment of TB disease. Additionally, DOT can aid in the early detection of adherence issues, adverse drug reactions, and worsening TB symptoms [11]. Previous estimates indicate that approximately 50% of patients on long-term treatment for chronic diseases often fail to adhere to their prescribed medication regimens in both developed and developing countries [12]. Several barriers hinder the effectiveness of DOT, including the inconvenience of daily in-person visits to health facilities, a shortage of health care workers, high transportation costs, TB-related stigma, and long patient waiting times at health facilities [13,14].

Digital adherence technologies have recently emerged as promising tools to address barriers to patient support and treatment monitoring at the health system, patient, and structural levels [15-18]. Video directly observed therapy (VDOT) is an innovative smartphone-based system that uses an app to record videos of medication intake, enabling remote monitoring of treatment adherence. VDOT reduces the need for frequent face-to-face meetings, thereby minimizing inconvenience to patients and lowering transportation costs [19,20]. Several observational studies have evaluated the feasibility and acceptability of VDOT, including 2 conducted in Africa [20-26]. Three randomized clinical trials (RCTs) in the United States, United Kingdom, and Moldova have assessed the efficacy of VDOT, demonstrating that it is feasible, acceptable, cost-saving, and convenient for patients [16,27-29]. A pilot study in Kenya found video-observed therapy to be both technically feasible and acceptable to patients and health care professionals [24]. Similarly, studies conducted among patients and health care providers in Uganda have shown that VDOT is feasible and acceptable for monitoring and supporting patients undergoing TB treatment [25,30,31]. A recent systematic review and meta-analysis found that VDOT is effective in improving medication adherence and bacteriological resolution compared with in-person DOT care [32]. However, none of the studies included in the review were conducted in Africa, as no RCTs comparing VDOT with in-person DOT have been published from the region. The aim of our study was to compare the effectiveness of VDOT and usual care directly observed therapy (UCDOT) in observing and monitoring treatment adherence among patients with TB in Uganda.

## Methods

### Ethical Considerations

This study was approved by the institutional review boards of the University of Georgia (ID PROJECT00000571), Makerere University (protocol 756), and the Uganda National Council for Science and Technology (HS656ES). All participants provided written informed consent in either English or Luganda, based on their language preference. The consent process included assurances regarding the privacy and confidentiality of the data collected during the study. Participants received approximately US $10 as compensation for transportation and the time spent participating in each visit.

### Trial Design

The “DOT Selfie” study was a parallel-group, open-label randomized controlled trial involving 144 adult patients with TB at selected treatment clinics in Kampala, Uganda. Participants were randomized into a control group (n=72), which received UCDOT, and an intervention group (n=72), which received VDOT for adherence monitoring. Enrollment into the RCT occurred between July 13, 2020, and follow-up continued through October 25, 2021. The details of the trial’s design and methods were described in our published protocol [33].

### Eligibility Criteria

Eligible participants were adults aged 18-65 years with a confirmed diagnosis of drug-susceptible TB in the new or retreatment category, who had initiated treatment within the past month and planned to reside in Kampala for the entire 6-month treatment period to facilitate close study follow-up. Participants were excluded if they could not speak and read either Luganda (the local dialect) or English, were too ill to withstand the study procedures at enrollment, or had a self-reported motor, visual, hearing, or cognitive disability that would hinder proper use of a smartphone. Additionally, patients residing in areas without cellular network coverage or access to electricity for charging their smartphones were excluded.

### Study Setting and Recruitment Procedures

**Figure 1:**
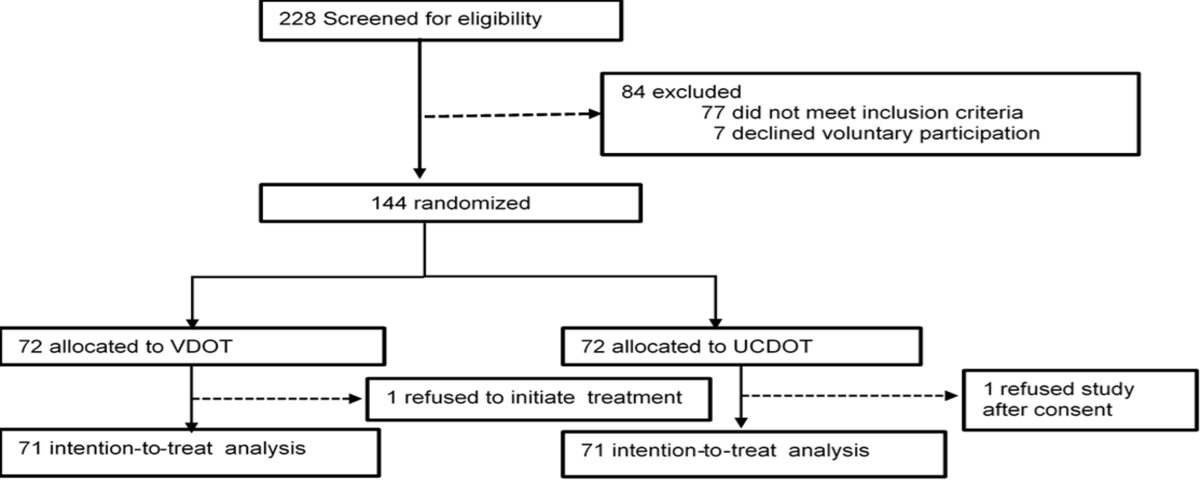
DOT Selfie participant flow diagram. UCDOT: usual care directly observed therapy; VDOT: video directly observed therapy.

The primary study site was the Lubaga TB clinic, with secondary recruitment sites being public clinics that provide free TB services in Kampala. All TB clinic sites are located about 10-15 km from the Kampala city center and collectively treat approximately 10,000 patients with TB annually. The study team worked closely with clinic nurses, who helped identify potentially eligible patients at the selected public clinic sites. Trained research assistants used a screening checklist to assess study eligibility, and then provided a brief description of the study. The details of the design and methods of the randomized controlled trial are described in our published protocol [33]. The baseline questionnaire was administered after consent, and a copy is provided in Multimedia Appendix 1. Participants were then randomized into the intervention or control study group. The study flow is shown in Figure 1.

### Randomization and Masking

We randomized 144 participants in a 1:1 ratio to receive either VDOT or UCDOT, as shown in Figure 1. We chose equal allocation to optimize the power of the study [34]. Randomization was stratified by sex to ensure equal representation of men and women in each study group [34]. Sex stratification was justified because sex is a known factor associated with adherence to TB treatment [34,35]. Permuted block randomization with block sizes of 4 and 6 was used to ensure a balanced assignment of participants to the intervention or control groups and to minimize the predictability of study arm assignment. Each block contained a specified number of randomly ordered treatment assignments, as recommended in clinical trial methods by Friedman et al [34]. The detailed process followed during randomization is published in the protocol elsewhere [33]. Given the nature of the intervention, participants and study staff were not masked to study group assignment. Study investigators were not blinded to allocation information due to ongoing scientific reviews and the possibility of making clinical decisions about participants’ treatment if it became necessary to withdraw them from the intervention. The trial statistician was blinded to study group assignment during the analyses.

### Description of the Enhanced VDOT System

**Figure 2:**
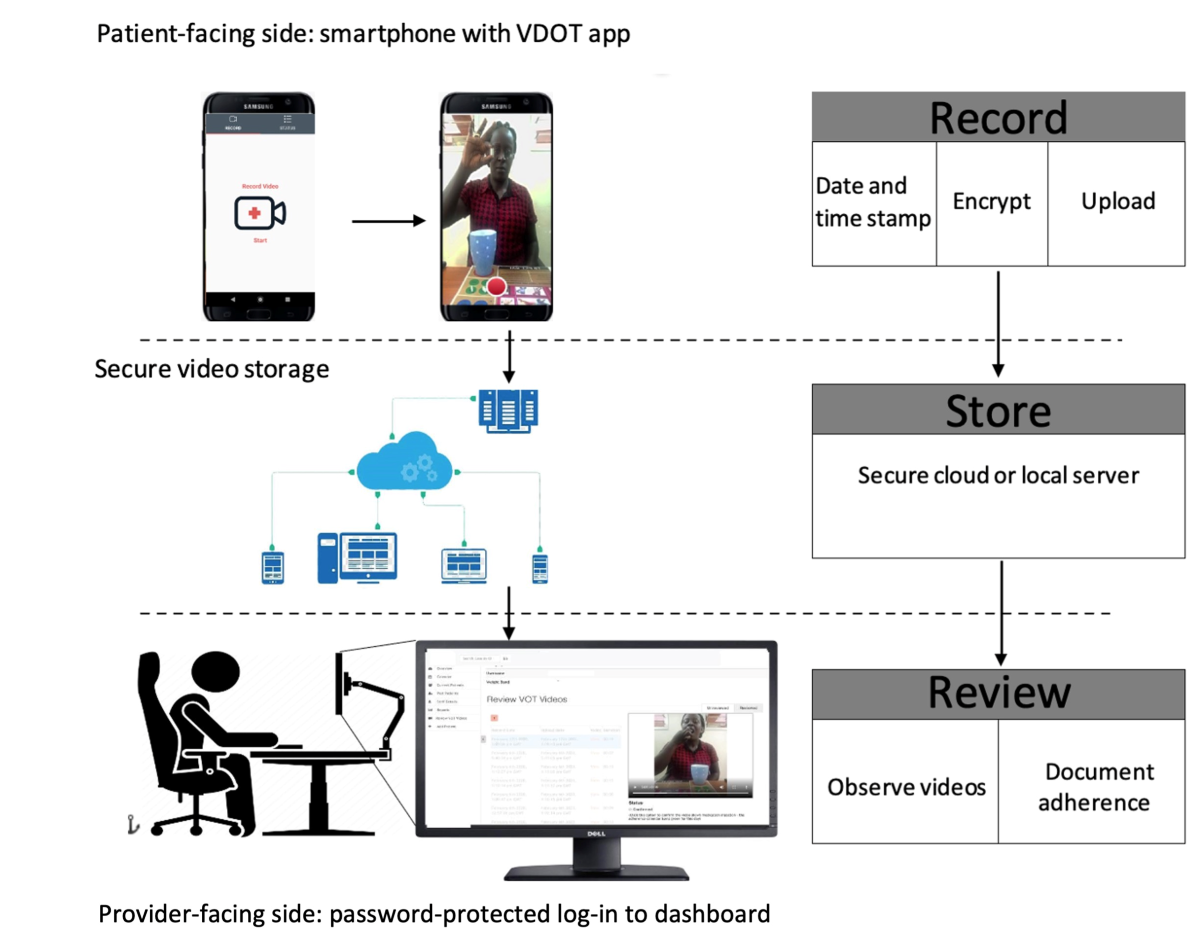
Schematic of the asynchronous video directly observed therapy system for monitoring tuberculosis treatment (adopted from Sekandi et al [33]).

VDOT is a licensed Health Insurance Portability and Accountability Act (HIPAA)–compliant system originally developed by SureAdhere Mobile Technology, an initiative that started at the University of California, San Diego [36]. It consists of 3 main components, as described previously by Garfein and colleagues [19] and in our published protocol paper [33]. The components include (1) the patient-facing part, which is the smartphone app used by the patient to record medication videos; (2) the secure cloud-based server, which stores the uploaded medication videos; and (3) the provider-facing part, which is the web-based browser accessed through a secure, password-protected log-in via a computer, laptop, or tablet to review the submitted videos and daily adherence reports [33]. At the end of each video recording, an automatic, encrypted, time-stamped video was uploaded to the secure cloud server for storage and playback (Figure 2). Upon submission, the video was removed from the smartphone, ensuring that patients could not retrieve it.

### Enhancement of the VDOT System for Contextual Adaptation

The VDOT system was adapted to the local Ugandan context in 3 ways. First, we expanded the English-based SMS text message reminders to include motivational messages encouraging patients to continue taking their medications and submitting videos. Second, we translated the SMS text messages into Luganda (a local dialect) to facilitate understanding for patients who did not speak English. The following are examples of the SMS text message reminders and motivational messages we used: “It’s time to take your pills and send a video” and “Taking your pills will help you to get better.” Third, an additional SMS text message reminder was sent automatically 8 hours after the standard SMS text message reminder, which was sent to participants at 6:00 AM, if a medication video had not been received in the VDOT system. After adapting the VDOT system to the local context, it was dubbed “DOT Selfie.”

### Description of the VDOT System

#### Description of Standard Procedures

The trial procedures are described in detail in our protocol paper, which is published elsewhere [33]. All participants had an initial face-to-face TB education session, regardless of the assigned study group. The session provided information on TB medications, including dosage, timing, the importance of taking daily pills as prescribed, and what to do in the event of a missed dose or lost pills. In addition, patients received information on cough hygiene behaviors, such as covering their cough to minimize the risk of spreading germs. Lastly, the education emphasized the importance of reporting any issues related to TB medications, including common side effects, adverse events, or new symptoms.

#### Intervention: Asynchronous Video Directly Observed Treatment

We used an asynchronous VDOT intervention package, which included (1) a loaner GSM (Global System for Mobile Communications) Itel P15 Android smartphone (model W5005) with a preinstalled VDOT app and a preassigned SIM card with a unique phone number; (2) a prepaid weekly internet data subscription of 350 MB at local telecom commercial rates; and (3) automated daily SMS text message reminders to take medications. Participants in the intervention group were enrolled in the VDOT system using a unique log-in personal identification number to set up an account and secure patient information in compliance with HIPAA [33]. The personal identification number also prevented access to the VDOT app by nonstudy participants. The app includes a video feature that allows patients to record themselves while swallowing their daily doses of medication. In addition, patients received weekly incentives equivalent to US $0.30 in the form of social bundles or airtime minutes, contingent on successfully submitting videos for 7 consecutive days. The weekly incentive was specifically linked to the VDOT intervention, meaning that participants randomized to this study group were eligible to receive the incentive. However, all participants, regardless of the study arm, received a transport refund and reimbursement for their time at each visit, equivalent to about US $10. On the day of enrollment, participants received detailed training on how to use the VDOT app to record and submit videos of medication intake. They also received an instructional guide with pictorial illustrations (pill mat) showing the systematic process of recording the videos, as well as a description of the most common side effects of TB medications. The “pill mat” served as a visual aid to facilitate easy reporting of medication side effects during the video recording session (see details in Multimedia Appendix 2).

At the clinic, a trained study staff member logged into the secure VDOT system via a computer or tablet to download and review patients’ daily videos, document medication adherence, and record any self-reported side effects. The study nurse also tracked missed video recordings and took follow-up actions as predefined in the protocol (see details in Multimedia Appendix 3). These actions included a follow-up call to assess missed doses, address side effects, and resolve any other issues as needed. Other secondary methods used to monitor adherence included pill counts and tracking prescription refills at the clinic.

#### Control: UCDOT

UCDOT was delivered according to routine practice under the Uganda National TB Program guidelines updated in 2017 [37]. The guidelines specify that the TB treatment supporter may be a health care worker, a trained workplace or community health worker, or anyone the patient chooses to observe them swallowing the tablets. The in-person interaction between the patient and the treatment supporter during treatment should occur at least five times a week, with observed medication doses documented in the treatment card. According to the guidelines, DOT services must be organized to accommodate the patients’ circumstances and provided as close to their homes as possible. Some patients may opt to take their medication at the health clinic if they live nearby. The patient and treatment supporter are expected to agree on a mutually convenient meeting place for treatment observation and support. However, there is considerable variation in how DOT is delivered across clinics and among patients in Kampala city. After a confirmed diagnosis, TB medications are prescribed, and patients return for biweekly refill visits during the first 2 months (intensive treatment phase). In the continuation phase, patients return monthly for refill visits and clinical evaluation.

In usual care, adherence is assessed using patients’ treatment cards, self-reports, and pharmacy prescription refills during routine clinic visits. The study research staff collaborated with TB clinic nurses or staff, community linkage facilitators, and treatment supporters to collect information on DOT. The research team combined data from all available sources and cross-validated it to the extent possible. Patients in the UCDOT study group were followed up for missed routine visits according to the National TB Program guidelines. This included a follow-up phone call if a patient missed a routine visit to refill a prescription, as well as addressing any issues, such as side effects, as needed. The COVID-19 pandemic caused some disruptions, resulting in unintended modifications to the routine delivery of UCDOT.

#### Follow-Up of the VDOT Group for Missed Videos and Study Visits

A predefined follow-up protocol was used to contact study participants in case of missed doses or videos (see Multimedia Appendix 3). The research staff made 2 phone call attempts within the first 24 hours of a missed video to establish the dosing history and determine the reason for the missed videos. If there was no response from the participant within 72 hours, the research team escalated the follow-up to a field visit to trace the participant at home or at their workplace. After 2 weeks of active follow-up with no success, the study team waited for the participant to return to the clinic for a routine visit. If the patient failed to show up for the scheduled clinic visit, the study team notified the national TB program staff to seek additional support. According to the standard WHO guidelines, a patient is declared lost to follow-up if they do not return to the clinic for their prescription refills for 2 consecutive months [38].

### Study Measurements

#### Primary Outcome

The primary outcome measure was adherence, defined as the fraction of expected doses observed (FEDO) over the months of prescribed TB treatment or by the end of the study following randomization. In the VDOT group, adherence was directly measured based on medication videos submitted and reviewed by the research staff to confirm medication ingestion. In the control UCDOT group, daily medication adherence was measured through self-reports. This was also supplemented with indirect measures, such as notes on the patients’ treatment card and prescription refill visits, as additional indicators of adherence. Study measurements were collected using a questionnaire at baseline and at follow-up during months 2, 4, and 6 to ensure close monitoring of all patients during treatment.

#### Secondary Outcomes

The secondary outcomes we selected are considered unfavorable outcomes and are also relevant indicators of performance for national TB programs. These include treatment completion, loss to follow-up, death, and treatment failure. The definitions of these outcomes were based on the WHO [38] and Uganda National TB Program guidelines [37]. Treatment completion was defined as completing all medication doses as prescribed by the TB clinic, as confirmed by the clinic records. Loss to follow-up was defined as an interruption in TB treatment for at least two consecutive months. Death was considered any death during the treatment period, regardless of the cause. Treatment failure was defined as a patient with TB whose sputum smear examination is positive at month 5 or later during TB treatment [38]. Treatment failure was difficult to ascertain due to incomplete clinic records. However, no cases of treatment failure were documented among the participants.

### Sample Size Estimation

The trial was powered at 80% to detect a 22% difference in medication adherence, the primary outcome, between the 2 groups: VDOT (85%) and UCDOT (63%), based on a 2-sided significance level of 5%. The adherence levels for the comparison groups were derived from previous DOT and VDOT studies conducted in Kampala, Uganda [25,39]. To achieve a chi-square test with a difference of 0.22 and an odds ratio of 0.30, the estimated total sample size was 124. We assumed an attrition rate of 14%, based on a randomized controlled trial comparing usual care and digital adherence interventions in Kenya, where the overall loss to follow-up rate was 11.7% (9.9% in UCDOT and 1.76% in VDOT) [40]. The calculated final sample size was 144 participants, with 72 per group. When calculating power and sample size, we treated the response as binary (0 or 1), with a cutoff value for nonadherence as the FEDO <80%, and adherence as the fraction ≥80%. This binary response variable for adherence was used in the final regression analysis to facilitate comparison with previous clinical trials [16]. Sample size tables were used to estimate precision, as described in detail elsewhere in our protocol paper [33]. All calculations were performed using the SAS statistical software package, version 9.4 (SAS Institute).

### Statistical Analyses

We conducted an intention-to-treat analysis according to the study group to which patients were originally randomized. Superiority was determined by a 22% difference in the proportion of patients achieving the primary outcome (63% vs 85%). VDOT treatment observation was classified as completed if patients’ medication doses were observed via video. UCDOT treatment observation was classified as completed if the patient reported that a designated treatment supporter observed the ingestion of prescribed doses. Univariate analysis was performed to describe the baseline characteristics of the study population. Multivariable logistic regression analysis was conducted to test for significant associations between the study groups (as the main exposure) and the primary binary outcome of adherence (no <80%, yes ≥80%). Age and HIV status were selected as potential confounders and were adjusted for in all models. Other covariates considered for adjustment during the primary analysis included education level, income, and smartphone ownership at baseline. We did not adjust for sex because randomization was stratified by this variable. Crude and adjusted risk ratios with 95% CIs are presented, and statistical significance was considered at *P* values less than .05.

## Results

### Overview

Of the 228 patients with confirmed TB who were screened, 144 met the eligibility criteria and were randomized into 1 of the 2 study groups, each with 72 participants. Two participants were excluded from the intention-to-treat analysis (n=142): 1 participant in the VDOT group refused to initiate TB treatment shortly after study enrollment, and another participant in the UCDOT group declined participation after initiating treatment. The 2 participants were included in the baseline analysis but excluded from any further analyses because they did not have the study outcomes of interest (Figure 1 and Multimedia Appendix 4 [41]). The median age of the participants was 34 (IQR 26-45) years. There was a significant difference in median age (*P*=.005) between the study groups, with participants in the UCDOT group being older; 120 of 144 (83.3%) participants owned a cell phone, while 53 of 144 (36.8%) had smartphones. Within the VDOT group, 29 of 72 (40%) owned a smartphone. Overall, 46 of 144 (31.9%) participants were HIV positive. All the measured baseline characteristics were balanced between the study groups, except for age (Table 1).

**Table 1:** Baseline characteristics of participants by study arm in Kampala, Uganda.

Variables		Total (N=144), n (%)	Video directly observed therapy arm (n=72), n (%)	Usual care directly observed therapy arm (n=72), n (%)	*P* value^a^
**Sex, n (%)**					
	Male	72 (50.0)	36 (50.0)	36 (50.0)	>.99
	Female	72 (50.0)	36 (50.0)	36 (50.0)	
Age (years), median (IQR)		34 (26-45)	29.5 (24-42)	38 (28-47)	*.005*
**Age category (years), n (%)**					
	18-24	27 (18.8)	19 (26.4)	8 (11.1)	*.02*
	25-34	48 (33.3)	27 (37.5)	21 (29.2)	
	35-44	30 (20.8)	10 (13.9)	20 (27.8)	
	45-65	39 (27.1)	16 (22.2)	23 (31.9)	
**Highest level of education, n (%)**					.12
	No education or primary	55 (38.2)	27 (37.5)	28 (38.9)	
	Secondary	64 (44.4)	28 (38.9)	36 (50.0)	
	Tertiary/university	25 (17.4)	17 (23.6)	8 (11.1)	
**Marital status, n (%)**					.33
	Currently married	55 (38.2)	23 (31.9)	32 (44.4)	
	Previously married	23 (16.0)	13 (18.1)	10 (13.9)	
	Never married	66 (45.8)	36 (50.0)	30 (41.7)	
**Currently employed, n (%)**					.61
	No	54 (37.5)	29 (40.3)	25 (34.7)	
	Yes	90 (62.5)	43 (59.7)	47 (65.3)	
Monthly personal income (US $^b^), median (IQR)		42.85 (14.29-85.71)	42.85 (14.29-114.30)	28.57 (11.43-85.71)	.27^c^
Monthly total household income (US $), median (IQR)		57.14 (28.57-142.86)	71.43 (28.57-157.14)	57.14 (28.57-114.23)	.56^c^
**Currently owns a cell phone, n (%)**					.50
	No	24 (16.7)	14 (19.4)	10 (13.9)	
	Yes	120 (83.3)	58 (80.6)	62 (86.1)	
**Currently owns a smartphone, n (%)**					.31
	No	67 (46.5)	29 (40.3)	38 (52.8)	
	Yes	53 (36.8)	29 (40.3)	24 (33.3)	
**HIV status, n (%)**					.55
	Positive	46 (31.9)	24 (33.3)	22 (30.6)	
	Negative	98 (68.1)	48 (66.7)	50 (69.4)	

^a^Significant values (*P*>.05) are presented in italics.

^b^US $1=3500 UGX (Uganda Shillings).

^c^Kruskal-Wallis test for differences in median.

### Patterns of Treatment Observation by VDOT and UCDOT Groups and by Sex

**Figure 3:**
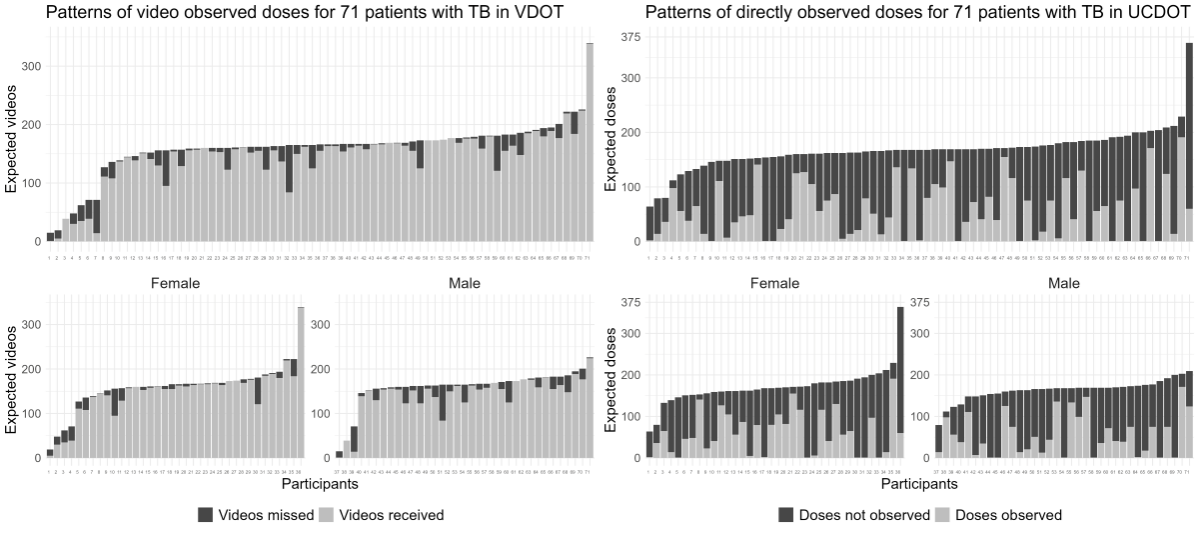
Patterns of treatment observation among participants in video directly observed therapy (VDOT) and usual care directly observed therapy (UCDOT). TB: tuberculosis.

A visual illustration of the patterns of treatment observation among study participants is shown in Figure 3. For the VDOT group, the light gray bars represent the videos received via the technology system, while the dark gray bars represent the videos that were not submitted (ie, missed). For the UCDOT group, the light gray bars show the doses that were observed (as reported by the patients) and the dark gray bars show the unobserved doses. We observed clear differences in the patterns of observation between the 2 study groups. However, when the same data are further stratified by sex, there are no obvious differences in the patterns of doses observed between men and women within each group.

### Median Differences in Expected and Observed Doses by Study Arm and Sex

There was a significant difference in the median number of doses observed between VDOT and UCDOT (Table 2). However, there were no significant differences in the number of doses when comparing male and female participants within the same study arm (Table 3).

**Table 2:** Median number of expected and observed doses by study arm.

Doses	Total, median (IQR)	Video directly observed therapy, median (IQR)	Usual care directly observed therapy, median (IQR)	*P* value
Expected videos/doses	167 (158-177)	166 (158-177)	168 (158-178)	.40
Videos/doses observed	118 (39-157)	156 (134-168)	48 (14-98)	<.001
Videos/doses not observed	37 (7-105)	7(2-23)	105 (66-151)	<.001

**Table 3:** Median number of expected and observed doses within study arms stratified by sex.

Arm and doses		Total, median (IQR)	Male, median (IQR)	Female, median (IQR)	*P* value	
**Usual care directly observed therapy** **arm**						
	Expected doses	168 (158-178)	168 (158-172)	168 (158-184)	.49	
	Doses observed	48 (14-98)	41 (14-86)	56 (12-99)	.61	
	Dose unobserved	105 (66-151)	116 (68-151)	104 (64-148)	.96	
**Video directly observed therapy** **arm**						
	Expected videos	166 (158-177)	165 (160-178)	166 (155-175)	.57	
	Video observed doses	156 (134-168)	154 (134-164)	160 (135-170)	.37	
	Videos missed, doses not observed	7 (2-23)	10 (3-26)	4 (2-14)	.16	

### Differences in Adherence Measured by the Fraction of Expected Doses Observed

We evaluated adherence using the FEDO, expressed as a percentage, as shown in Table 4. The overall median FEDO was 80% (IQR 30%-100%), but the median adherence in the VDOT arm was 100% (IQR 80%-100%), which was significantly higher (*P*<.001) than that of the UCDOT group. When using a cutoff of ≥80% for FEDO to define optimal adherence and <80% for nonadherence, only 63 of 142 (44.4%) participants, on average, achieved optimal adherence with their expected doses observed. However, in the VDOT group, 56 of 71 (79%) participants achieved an optimum FEDO, which was significantly higher (*P*<.001) than in the UCDOT group. The median FEDO did not significantly differ by sex in either study arm (*P*=.16 for VDOT and *P*=.96 for UCDOT).

**Table 4:** Median adherence based on FEDO^a^ by study arm.

Variables			Total		Video directly observed therapy		Usual care directly observed therapy		*P* value			
Adherence (%), median FEDO (IQR)			80 (30-100)		100 (80-100)		30 (10-60)		<.001			
**Adherence, with FEDO as a binary outcome (yes ≥80% and no <80%), n (%)**										<.001		
	Yes, n (%)	63 (44)		56 (79)		7 (10)						
	No, n (%)	79 (66)		15 (21)		64 (90)						
	Total, n	142		71		71						

^a^FEDO: fraction of expected doses observed.

### Factors Associated With Treatment Observation as a Measure of Adherence

We performed a multivariable logistic regression analysis to evaluate the factors associated with optimal adherence levels (≥80% doses observed), as measured by the FEDO, with the results presented in Table 5. The results from the unadjusted regression models indicated that being in the VDOT group and identifying as Muslim were independent factors significantly (*P*=.03) associated with achieving adherence of ≥80%. We found that participants in the VDOT group were 8.4 times more likely to have ≥80% of their expected doses observed compared with those in the UCDOT group, after adjusting for confounding factors such as age, religion, current cell phone ownership, and HIV status. Current cell phone ownership was significantly (*P*=.04) associated with reaching the 80% adherence level, based on expected doses observed, compared with those who did not own a cell phone, after adjusting for the study group and other covariates.

**Table 5:** Factors associated with treatment adherence, as measured by FEDO^a^, among study participants in Kampala, Uganda^b^.

Variables		Unadjusted risk ration (95% CI)	*P* value	Adjusted risk ration (95% CI)	*P* value
**Arm**					
	Usual care directly observed therapy	1.00	N/A^c^	1.00	N/A
	Video directly observed therapy	*8 (3.92 to 16.33)*	*<.001*	*8.41 (4.16 to 17)*	*<.001*
**Sex**					
	Male	1.00	N/A	1.00	N/A
	Female	1 (0.69 to 1.45)	.99	0.9 (0.61 to 1.32)	.59
**Age category (years)**					
	45-65	1.00	N/A	1.00	N/A
	35-44	0.74 (0.38 to 1.45)	.38	1.02 (0.6 to 1.74)	.95
	25-34	1.18 (0.72 to 1.93)	.50	1.06 (0.73 to 1.54)	.75
	18-24	1.46 (0.89 to 2.41)	.14	1.03 (0.69 to 1.52)	.89
**Highest education level**					
	No education or primary	1.00	N/A	1.00	N/A
	Secondary	0.95 (0.63 to 1.41)	.78	0.88 (0.62 to 1.25)	.46
	Tertiary/University	0.90 (0.52 to 1.57)	.71	0.6 (0.36 to 1.01)	.06
**Marital status**					
	Married	1.00	N/A	1.00	N/A
	Previously married	1.17 (0.69 to 2)	.56	1 (0.63 to 1.6)	.99
	Never married	1.13 (0.75 to 1.72)	.56	0.99 (0.74 to 1.31)	.93
**Religion**					
	Catholic	1.00	N/A	1.00	N/A
	Protestant	1.59 (0.93 to 2.72)	.09	1.06 (0.7 to 1.61)	.77
	Muslim	*1.75 (1.04 to 2.93)*	*.03*	1.29 (0.87 to 1.91)	.20
	Pentecostal/Seventh Day Adventist/Other	1.59 (0.92 to 2.76)	.10	1.01 (0.7 to 1.48)	.94
**Currently own a cell phone**					
	No	1.00	N/A	1.00	N/A
	Yes	1.22 (0.7 to 2.12)	.48	*1.62 (1.02 to 2.57)*	*.04*
**HIV status**					
	HIV negative	1.00	N/A	1.00	N/A
	HIV positive	0.87 (0.57 to 1.33)	.52	0.8 (0.56 to 1.14)	.22

^a^FEDO: Fraction of expected doses observed.

^b^Significant values (*P*>.05) are presented in italics.

^c^N/A: not applicable.

In the intention-to-treat analysis of 142 participants, the secondary TB outcomes (Table 6) evaluated showed treatment completion at 90.1% (128/142) for all participants, with significantly higher completion rates in the VDOT group compared with the UCDOT group. The rate of loss to follow-up was 6.3% (9/142) overall, but 7 of 71 (10%) participants in the UCDOT group were lost to follow-up, compared with 2 of 71 (3%) in the VDOT group. Overall, there were 5 deaths; 4 of these were HIV coinfected and 3 were male (see details in Table 7).

**Table 6:** Secondary tuberculosis outcomes.

Outcome	Total (n=142)	Video directly observed therapy (n=71)	Usual care directly observed therapy (n=71)	*P* value
Treatment completion, n (%)	128 (90.1)	67 (94.4)	61 (85.9)	.04
Lost to follow-up, n (%)	9 (6.3)	2 (2.8)	7 (9.9)	.02
Death, n (%)	5 (3.5)	2 (2.8)	3 (4.2)	.16
Treatment failure at 5 months, n	0	0	0	N/A^a^

^a^N/A: not applicable.

**Table 7:** Summary of deaths.

Participants	Study arm	Sex	Age (years)	HIV status	Number of treatment doses taken
1	VDOT^a^	Male	29	Positive	56
2	VDOT	Female	28	Positive	84
3	UCDOT^b^	Male	40	Positive	112
4	UCDOT	Female	54	Positive	84
5	UCDOT	Male	62	Negative	128

^a^VDOT: video directly observed therapy.

^b^UCDOT: Usual care directly observed therapy.

### Reasons for Missed Video Submissions in the VDOT Group

Based on the standard 6-month regimen of 168 doses and the enrollment date, we expected a total of 11,928 videos. Of these, 11,041 (92.56%) were successfully submitted by participants included in the intention-to-treat analysis over the study period. Of the 887 missed videos (7.44%), the most common reason reported by participants was a lack of a charged phone battery, accounting for 183 of the 887 (20.6%) missed videos. Technical or technology issues accounted for 450 (50.7%) of the missed videos. Patient-related issues accounted for 348 (39.2%) missed videos, with the most common reason (136/887, 15.3%) being forgetting to record the videos while swallowing the medication. Additional reasons for missed videos are provided in Table 8.

**Table 8:** Reasons for missed videos among VDOT^a^ study participants in Kampala, Uganda.

Reasons for missed videos				Patients reporting (n=212), n		Videos missed (n=887), n (%)
**Technical or technology-related issues**						
	Phone battery is not charged		39		183 (20.6)	
	Phone stolen/lost		6		98 (11.0)	
	VDOT app errors		23		72 (8.1)	
	Reported lack of internet connection		22		63 (7.1)	
	Phone malfunction		7		18 (2.0)	
	Failed to use the app, needed retraining		12		16 (1.8)	
**Patient-related issues**						
		Took medications but forgot to record videos		21		136 (15.3)
		Traveled to a location that has no electricity		16		74 (8.3)
		Too ill to record the video but took medications		7		51 (5.7)
		Too busy to record videos		13		40 (4.5)
		Location is not convenient to record		7		24 (2.7)
		Ran out of tuberculosis medications		16		23 (2.6)
**Other not classified**						
		No reason specified		23		89 (10.0)

^a^VDOT: video directly observed therapy.

## Discussion

### Principal Findings

In this study, we found that VDOT was significantly more effective in observing medication ingestion and monitoring adherence among patients with TB. Our findings align with a recent systematic review and meta-analysis that included studies conducted in Australia, China, Moldova, the United Kingdom, and the United States. The review concluded that monitoring treatment using VDOT was significantly associated with increased adherence and better microbiological outcomes compared with UCDOT [32]. Additionally, we found that cell phone ownership was significantly associated with a higher level of observed doses, regardless of the study group. Although VDOT was effective, some participants occasionally failed to submit their expected videos of medication intake. The most common reasons for missed videos were lack of a charged phone battery, forgetting to record the medication event, and loss of the smartphone. This finding is consistent with results from our previous pilot study conducted in Kampala, Uganda [25]. To our knowledge, this study is one of the few randomized controlled trials conducted to evaluate the effectiveness of VDOT in Uganda and Africa. More studies are needed to assess the effectiveness of VDOT in rural African contexts and other low-resource settings. Additionally, it is important to identify which subgroups of patients are most likely to benefit from this technology.

There are a few published randomized controlled trials that have compared various forms of VDOT intervention packages with in-person DOT thus far [16,28,29]. Story and colleagues [16] published the first multicenter trial evaluating the efficacy of video-observed treatment compared with usual care in 2019. The study was conducted in the United Kingdom and involved a larger sample of 226 patients from 22 centers. The sample was also highly diverse, with 58% of participants having a history of drug and alcohol abuse, homelessness, and incarceration. In the UK study, 70% of patients on VDOT achieved optimal (≥80%) scheduled observations during the first 2 months, compared with 31% in the UCDOT group. The difference observed in the VDOT arm could be attributed to the difference in the timing of the measurement of the primary outcome, which was 2 months versus 6 months after randomization. The Moldova trial by Ravenscroft and colleagues [28] compared patients assigned to VDOT and clinic-based DOT. The study measured the primary outcome as the observed medication adherence every 2 weeks for the entire 6 months of treatment. The study concluded that VDOT significantly decreased nonadherence compared with standard DOT and reduced the time and money patients spent during their treatment [28]. Although there were some differences in the delivery of the VDOT intervention package and the measurement of the primary outcome, the conclusions on the effectiveness of VDOT are quite similar across studies.

The UCDOT group had a low level (21/71, 30%) of reported observed doses of medication over the 6 months of treatment. This result is very similar to the findings from the UK trial, despite differences in the study populations, delivery of DOT, and treatment durations [16]. This finding further reinforces the fact that in-person DOT is generally difficult to implement, particularly in low-resource settings [39,42]. A Cochrane review of 11 clinical trials concluded that TB cure and treatment completion rates were not improved by DOT [13]. In South Africa, a recent study found that only about 25% of patients with TB received formal DOT support, either home-based or clinic-based [43].

Cell phone ownership was significantly associated with achieving optimal observation of the expected doses of medications, regardless of the study group. Moreover, we found that 120 of 142 (84.5%) patients owned a cell phone, while 53 of 142 (37.3%) had personal smartphones at baseline. This finding suggests that having access to a cell phone could serve as an indirect facilitator of adherence support for patients on treatment. In African settings, smartphone ownership and cellular networks are rapidly proliferating, presenting opportunities to leverage modern technology to improve health care and service delivery. According to the National Institutes of Health, “there is a need to stimulate research utilizing mHealth tools aimed at the improvement of adherence to treatment, effective patient-provider communication, and self-management of chronic diseases in underserved populations” [44]. There is increased utility and effectiveness of accessible mobile technologies in enhancing the monitoring of patients with chronic diseases, which can be attributed to the affordability and reliability of smartphones in both high- and low-income settings [45,46].

The main secondary outcomes, including treatment completion and loss-to-follow-up, were statistically better in the VDOT group compared with the UCDOT group. These findings are consistent with the conclusions of a recent systematic review and meta-analysis [32]. The notably higher percentage of TB/HIV coinfected individuals among the deaths aligns with epidemiologic trends [2]. Treatment failure is an important outcome, but it is often difficult to ascertain, especially with follow-up periods of less than 1 year. To determine treatment failure, we used the records at the end of treatment at 6 months, although it should be routinely assessed at 5 months. We relied on the TB clinic records of sputum results at month 5 because our study protocol included only follow-up visits at months 2, 4, and 6. The study team did not have direct control over the standard clinical evaluations that occur in routine TB management. The clinic records were grossly deficient; therefore, we cannot conclusively state that there were no treatment failures. Larger studies are needed to compare these outcomes across the groups to ascertain the true differences.

### Reasons for Failure to Submit Videos

Overall, technical and technology-related issues were the most commonly cited reasons for failure to submit videos. The lack of a charged phone battery was the single most common reason for failure to submit videos, as sometimes phones turned off during video recording. This finding is consistent with results from our pilot study in Kampala [25,47]. To minimize the phone battery issue, patients were trained and encouraged to ensure that their phones were always charged at night or before recording videos. The loss of a smartphone occurred among a few participants, resulting in a substantial number of missed videos. The study team mitigated the disruption in data collection by replacing lost phones as soon as they were made aware. Missing adherence records were filled using self-reports from patients or clinic records on prescription refills. However, the self-report method falls short because it lacks an objective way to validate the doses taken [48].

The main patient-related reasons that mostly prevented the submission of videos included forgetting to record the medication intake, traveling to locations without access to electricity to charge the phone, or being too ill to record videos. These findings were similar to those reported in our first pilot study [25] and in other studies elsewhere [19,26]. Typically, when patients travel to rural areas in Uganda, there is a high likelihood that they will not have access to electricity, further complicating adherence monitoring. When videos were missed due to travel, the research team followed up to gather the missing information using self-reported adherence. A small number of patients reported a lack of a convenient place to record videos, which was a concern similar to our previous study [25]. This could suggest an underlying stigma associated with TB treatment. However, a study done in Turkey, which compared the levels of stigma among patients with TB using in-person DOT versus VDOT, concluded that patients who received VDOT reported experiencing less stigma [49]. The role of VDOT in mitigating stigma among patients with TB warrants further research, especially in diverse contexts.

### Strengths in the Context of Prior Work

First, we evaluated the enhanced VDOT intervention, which is the only digital adherence technology offering remote visual observation of doses taken, making it comparable to the UCDOT. Other commonly used digital adherence technologies for TB monitoring, such as 99 DOTS and digital or smart pillboxes, only provide indirect signals as proxies for doses of medication intake [18]. Second, we successfully evaluated a culturally adapted VDOT in a randomized controlled trial (RCT) within the context of a low-income setting. In our study, we demonstrated that more than one-third of the participants owned a personal smartphone, and these numbers are growing globally. Third, we conducted the study in Kampala, Uganda, where the TB rates are estimated at 250 cases per 100,000, which is much higher than in the majority of settings where VDOT has been previously evaluated [16,19,29]. Fourth, the study facilitated the delivery of a patient-centered approach that was more convenient and less intrusive during the critical period of the COVID-19 pandemic. This also catered to the public health mitigation measures aimed at curtailing the transmission of COVID-19. Lastly, the RCT results generated in our study provide a basis for the systematic evaluation of the implementation process of VDOT. Although there is no gold standard for using digital adherence technologies to monitor TB treatment, our study adds to the mounting evidence that supports the digital observation of pill-taking as an alternative to in-person DOT in usual care.

### Study Limitations

First, the study was conducted during the COVID-19 pandemic; therefore, some unintended disruptions in the health system could have altered the practical delivery of usual DOT care in Kampala, Uganda. This may have led to an overestimation of the effect of VDOT. However, a study comparing VDOT and UCDOT before and during COVID-19 found significantly higher adherence to TB treatment when using VDOT during both periods [50]. It is possible that adherence in the UCDOT group could have been greater if not for social distancing mandates and lockdowns. The potential effect of the pandemic period may limit the generalizability of our findings. Second, our study did not specifically measure clinical endpoints, such as sputum conversion at follow-up visits. Although we attempted to extract this information from routine clinic records, the data were incomplete. Third, our study was conducted in an urban setting, which may limit its generalizability to rural settings due to differences in key factors such as cellular network availability, internet coverage, and access to electricity for charging phone batteries. Lastly, the enhanced VDOT intervention included multiple components; however, this study was not designed to isolate the effects of specific components that may have contributed to the differences in adherence observed. Future studies could use a factorial design to assess the impact of different intervention components. There are cross-cutting limitations common to all digital health technologies, such as technology infrastructure, cost, and access to smartphones, which will impact adoption, scale-up, and sustainability, particularly in low-income settings [18].

### Considerations for Future Adoption, Scale-Up, and Sustainability of VDOT

We proposed several considerations for the broader adoption, scale-up, and sustainability of VDOT in low-resource settings. Our findings support the need to carefully address the known facilitators and barriers to VDOT acceptability, such as those highlighted in the qualitative exit study of patients who used VDOT in Uganda [30]. First, VDOT requires upfront investment in the health system and local technology infrastructure to facilitate scale-up. A robust system would include the hardware—computers or smartphones—and the software—access to the internet, cellular networks, and maintenance of the VDOT system. There is also an overarching need for adequate access to electricity. Second, national TB programs could leverage public-private partnerships with entities such as telecommunication companies that manage vital resources such as internet coverage for sustainability. Third, standardized training for health providers is essential to facilitate the effective use and adoption of digital technology. Fourth, there is a need to integrate the VDOT system into the general health management information system to ensure interoperability and the timely use of adherence outcome data to improve patient care. The VDOT system could eventually be optimized for monitoring treatment adherence across multiple diseases, such as HIV/AIDS, diabetes, and hypertension. Lastly, patients would need basic training in the use of the VDOT app to ensure ease of use. In our qualitative exit interviews with users, training in the use of the smartphone app was cited as a major facilitator of ease of use and acceptability for VDOT [30]. In general, we expect that health providers and patients will grow more experienced and comfortable with technology over time.

### Ethical-Legal Issues and Areas for Future Research

Confidentiality, privacy, and stigma concerns are overarching issues that must be carefully addressed to alleviate fears of intentional or unintended disclosure of a patient’s disease status. The flexibility of making video recordings at the patient’s desired time and place greatly enhances privacy [51]. In a VDOT study conducted in the United States and Mexico, patients agreed that VDOT promotes autonomy and a sense of control over their health [19]. The ethical concerns related to digital technologies can vary widely, particularly in relation to collecting, securely storing, and accessing videos that contain personally identifiable information, such as patients’ faces. These concerns may also have legal and sociocultural contexts that need to be considered [52]. A recent qualitative study focusing on digital health technologies in sub-Saharan Africa concluded that threats to scale-up include the lack of buy-in from both patients and providers, insufficient human resources and local capacity, inadequate governmental support, overly restrictive regulations, and a lack of focus on cybersecurity and data protection [53]. Further research is needed to evaluate the impact of VDOT on patient-centered outcomes, such as satisfaction, quality of life, user engagement with the technology, and retention to prevent dropout. Other important areas of research include the effect of VDOT on health system workflows and workload, as well as its costs and cost-effectiveness compared with usual care.

### Conclusions

The enhanced VDOT was more effective in increasing adherence observation to treatment than UCDOT among patients with TB in Uganda. This evidence supports the potential of digital technologies to improve monitoring and support of treatment adherence in high TB burden settings with limited human resources. Further research is needed to evaluate which subgroups are most likely to benefit from this technology.

## Appendix

Multimedia Appendix 1 Baseline questionnaire.

Multimedia Appendix 2 Video directly observed therapy pill mat.

Multimedia Appendix 3 Video directly observed therapy follow-up protocol.

Multimedia Appendix 4 CONSORT E-HEALTH checklist. CONSORT: Consolidated Standards of Reporting Trials.

## Abbreviations

DOT: directly observed therapy
FEDO: fraction of expected doses observed
GSM: Global System for Mobile Communications
HIPAA: Health Insurance Portability and Accountability Act
MDR-TB: multidrug-resistant tuberculosis
RCT: randomized clinical trial
TB: tuberculosis
UCDOT: usual care directly observed therapy
VDOT: video directly observed therapy
WHO: World Health Organization

## References

[1] Uplekar M, Weil D, Lonnroth K, Jaramillo E, Lienhardt C, Dias HM, Falzon D, Floyd K, Gargioni G, Getahun H, Gilpin C, Glaziou P, Grzemska M, Mirzayev F, Nakatani H, Raviglione M (2015). WHO's new end TB strategy. The Lancet.

[2] World Health Organization (WHO) (2023). Global Tuberculosis Report. WHO.

[3] Dick J, Lombard C (1997). Shared vision--a health education project designed to enhance adherence to anti-tuberculosis treatment. Int J Tuberc Lung Dis.

[4] Toczek A, Cox H, du Cros P, Cooke G, Ford N (2013). Strategies for reducing treatment default in drug-resistant tuberculosis: systematic review and meta-analysis. Int J Tuberc Lung Dis.

[5] Zegeye A, Dessie Getnet, Wagnew Fasil, Gebrie Alemu, Islam Sheikh Mohammed Shariful, Tesfaye Bekele, Kiross Dessalegn (2019). Prevalence and determinants of anti-tuberculosis treatment non-adherence in Ethiopia: a systematic review and meta-analysis. PLoS One.

[6] Gube A, Debalkie Megbaru, Seid Kalid, Bisete Kiberalem, Mengesha Asfaw, Zeynu Abubeker, Shimelis Freselam, Gebremeskel Feleke (2018). Assessment of Anti-TB drug nonadherence and associated factors among TB patients attending TB clinics in Arba Minch governmental health institutions, Southern Ethiopia. Tuberc Res Treat.

[7] Adane A, Alene Kefyalew Addis, Koye Digsu Negese, Zeleke Berihun Megabiaw (2013). Non-adherence to anti-tuberculosis treatment and determinant factors among patients with tuberculosis in northwest Ethiopia. PLoS One.

[8] Salari N, Kanjoori Amir Hossein, Hosseinian-Far Amin, Hasheminezhad Razie, Mansouri Kamran, Mohammadi Masoud (2023). Global prevalence of drug-resistant tuberculosis: a systematic review and meta-analysis. Infect Dis Poverty.

[9] World Health Organization (WHO) (2017). Guidelines for treatment of drug-susceptible tuberculosispatient care, 2017 update. WHO.

[10] Nahid P, Dorman Susan E, Alipanah Narges, Barry Pennan M, Brozek Jan L, Cattamanchi Adithya, Chaisson Lelia H, Chaisson Richard E, Daley Charles L, Grzemska Malgosia, Higashi Julie M, Ho Christine S, Hopewell Philip C, Keshavjee Salmaan A, Lienhardt Christian, Menzies Richard, Merrifield Cynthia, Narita Masahiro, O'Brien Rick, Peloquin Charles A, Raftery Ann, Saukkonen Jussi, Schaaf H Simon, Sotgiu Giovanni, Starke Jeffrey R, Migliori Giovanni Battista, Vernon Andrew (2016). Official American Thoracic Society/Centers for Disease Control and Prevention/Infectious Diseases Society of America Clinical Practice Guidelines: treatment of drug-susceptible tuberculosis. Clin Infect Dis.

[11] World Health Organization (WHO) (2017). Guidelines for treatment of drug-susceptible tuberculosis and patient care ( 2017 update). WHO.

[12] World Health Organization (WHO) (2003). Adherence to Long-term Therapies Evidence for Action.

[13] Karumbi J, Garner P (2015). Directly observed therapy for treating tuberculosis. Cochrane Database Syst Rev.

[14] Yang Wei-Teng, Gounder Celine R, Akande Tokunbo, De Neve Jan-Walter, McIntire Katherine N, Chandrasekhar Aditya, de Lima Pereira Alan, Gummadi Naveen, Samanta Santanu, Gupta Amita (2014). Barriers and delays in tuberculosis diagnosis and treatment services: does gender matter?. Tuberc Res Treat.

[15] Holzman Samuel B, Atre Sachin, Sahasrabudhe Tushar, Ambike Sunil, Jagtap Deepak, Sayyad Yakub, Kakrani Arjun Lal, Gupta Amita, Mave Vidya, Shah Maunank (2019). Use of smartphone-based video directly observed therapy (vDOT) in tuberculosis care: single-arm, prospective feasibility study. JMIR Form Res.

[16] Story A, Aldridge Robert W, Smith Catherine M, Garber Elizabeth, Hall Joe, Ferenando Gloria, Possas Lucia, Hemming Sara, Wurie Fatima, Luchenski Serena, Abubakar Ibrahim, McHugh Timothy D, White Peter J, Watson John M, Lipman Marc, Garfein Richard, Hayward Andrew C (2019). Smartphone-enabled video-observed versus directly observed treatment for tuberculosis: a multicentre, analyst-blinded, randomised, controlled superiority trial. Lancet.

[17] Cook R, Lamont Tara, Martin Rosie, NIHR Dissemination Centre (2019). Smartphones can improve adherence rates for TB treatment. BMJ.

[18] Subbaraman R, de Mondesert Laura, Musiimenta Angella, Pai Madhukar, Mayer Kenneth H, Thomas Beena E, Haberer Jessica (2018). Digital adherence technologies for the management of tuberculosis therapy: mapping the landscape and research priorities. BMJ Glob Health.

[19] Garfein R, Collins K, Muñoz F, Moser K, Cerecer-Callu P, Raab F, Rios P, Flick A, Zúñiga M L, Cuevas-Mota J, Liang K, Rangel G, Burgos J L, Rodwell T C, Patrick K (2015). Feasibility of tuberculosis treatment monitoring by video directly observed therapy: a binational pilot study. Int J Tuberc Lung Dis.

[20] Krueger K, Ruby D, Cooley P, Montoya B, Exarchos A, Djojonegoro B M, Field K (2010). Videophone utilization as an alternative to directly observed therapy for tuberculosis. Int J Tuberc Lung Dis.

[21] Mirsaeidi M, Farshidpour Maham, Banks-Tripp Deborah, Hashmi Sarah, Kujoth Carrie, Schraufnagel Dean (2015). Video directly observed therapy for treatment of tuberculosis is patient-oriented and cost-effective. Eur Respir J.

[22] DeMaio J, Schwartz L, Cooley P, Tice A (2001). The application of telemedicine technology to a directly observed therapy program for tuberculosis: a pilot project. Clin Infect Dis.

[23] Gassanov M, Feldman Linda J, Sebastian Adrian, Kraguljac Marnie J, Rea Elizabeth, Yaffe Barbara (2013). The use of videophone for directly observed therapy for the treatment of tuberculosis. Can J Public Health.

[24] Hoffman J, Cunningham Janice R, Suleh Andrew J, Sundsmo Aaron, Dekker Debra, Vago Fred, Munly Kelly, Igonya Emmy Kageha, Hunt-Glassman Jonathan (2010). Mobile direct observation treatment for tuberculosis patients: a technical feasibility pilot using mobile phones in Nairobi, Kenya. Am J Prev Med.

[25] Sekandi J, Buregyeya Esther, Zalwango Sarah, Dobbin Kevin K, Atuyambe Lynn, Nakkonde Damalie, Turinawe Julius, Tucker Emma G, Olowookere Shade, Turyahabwe Stavia, Garfein Richard S (2020). Video directly observed therapy for supporting and monitoring adherence to tuberculosis treatment in Uganda: a pilot cohort study. ERJ Open Res.

[26] Nguyen T, Pham Minh Tam, Nguyen Thi Loi, Nguyen Viet Nhung, Pham Duc Cuong, Nguyen Binh Hoa, Fox Greg James (2017). Video directly observed therapy to support adherence with treatment for tuberculosis in Vietnam: a prospective cohort study. Int J Infect Dis.

[27] Hayward AG, Garber E (2014). TB Reach 5: to compare the efficacy of video observed treatment (VOT) versus directly observed treatment (DOT) in supporting adherence in patients with active tuberculosis. ISRCTN.

[28] Ravenscroft Luke, Kettle Stewart, Persian Ruth, Ruda Simon, Severin Lilian, Doltu Svetlana, Schenck Benjamin, Loewenstein George (2020). Video-observed therapy and medication adherence for tuberculosis patients: randomised controlled trial in Moldova. Eur Respir J.

[29] Burzynski J, Mangan Joan M, Lam Chee Kin, Macaraig Michelle, Salerno Marco M, deCastro B Rey, Goswami Neela D, Lin Carol Y, Schluger Neil W, Vernon Andrew, eDOT Study Team (2022). In-person vs electronic directly observed therapy for tuberculosis treatment adherence: a randomized noninferiority trial. JAMA Netw Open.

[30] Sekandi J, McDonald Adenike, Nakkonde Damalie, Zalwango Sarah, Kasiita Vicent, Kaggwa Patrick, Kakaire Robert, Atuyambe Lynn, Buregyeya Esther (2023). Acceptability, usefulness, and ease of use of an enhanced video directly observed treatment system for supporting patients with tuberculosis in Kampala, Uganda: explanatory qualitative study. JMIR Form Res.

[31] Sekandi J, Kasiita Vicent, Onuoha Nicole Amara, Zalwango Sarah, Nakkonde Damalie, Kaawa-Mafigiri David, Turinawe Julius, Kakaire Robert, Davis-Olwell Paula, Atuyambe Lynn, Buregyeya Esther (2021). Stakeholders' perceptions of benefits of and barriers to using video-observed treatment for monitoring patients with tuberculosis in Uganda: exploratory qualitative study. JMIR Mhealth Uhealth.

[32] Truong C, Tanni Kaniz A, Qian Jingjing (2022). Video-observed therapy versus directly observed therapy in patients with tuberculosis. Am J Prev Med.

[33] Sekandi J, Onuoha Nicole Amara, Buregyeya Esther, Zalwango Sarah, Kaggwa Patrick Evans, Nakkonde Damalie, Kakaire Robert, Atuyambe Lynn, Whalen Christopher C, Dobbin Kevin K (2021). Using a mobile health intervention (DOT Selfie) with transfer of social bundle incentives to increase treatment adherence in tuberculosis patients in Uganda: protocol for a randomized controlled trial. JMIR Res Protoc.

[34] Friedman LM, Furberg CD, DeMets DL, Reboussin DM (2015). Fundamentals of Clinical Trials.

[35] Feng J, Huang S-f, Ting W-y, Chen Y-c, Lin Y-y, Huang R-m, Lin C-h, Hwang J-j, Lee J-j, Yu M-c, Yu K-w, Lee Y-c, Su W-j (2012). Gender differences in treatment outcomes of tuberculosis patients in Taiwan: a prospective observational study. Clin Microbiol Infect.

[36] SureAdhere Mobile Technology, Inc.

[37] (2017). Manual for management and control of tuberculosis and leprosy (3rd edition). Ministry of Health, Republic of Uganda.

[38] World Health Organization (WHO) (2013). Definitions and reporting framework for tuberculosis. WHO.

[39] Hassard Sempeera, Ronald Anguzu, Angella Kawooya (2017). Patient attitudes towards community-based tuberculosis DOT and adherence to treatment in an urban setting; Kampala, Uganda. Pan Afr Med J.

[40] Yoeli E, Rathauser Jon, Bhanot Syon P, Kimenye Maureen K, Mailu Eunice, Masini Enos, Owiti Philip, Rand David (2019). Digital health support in treatment for tuberculosis. N Engl J Med.

[41] Eysenbach Gunther, CONSORT-EHEALTH Group (2011). CONSORT-EHEALTH: improving and standardizing evaluation reports of Web-based and mobile health interventions. J Med Internet Res.

[42] Kisambu J, Nuwaha F, Sekandi J N (2014). Adherence to treatment and supervision for tuberculosis in a DOTS programme among pastoralists in Uganda. Int J Tuberc Lung Dis.

[43] Howell E, Kigozi N Gladys, Heunis J Christo (2018). Community-based directly observed treatment for TB patients to improve HIV services: a cross-sectional study in a South African province. BMC Health Serv Res.

[44] (2016). mHealth tools for individuals with chronic conditions to promote effective patient-provider communication, adherence to treatment and self-management Internet. National Institutes of Health.

[45] Falzon D, Timimi Hazim, Kurosinski Pascal, Migliori Giovanni Battista, Van Gemert Wayne, Denkinger Claudia, Isaacs Chris, Story Alistair, Garfein Richard S, do Valle Bastos Luis Gustavo, Yassin Mohammed A, Rusovich Valiantsin, Skrahina Alena, Van Hoi Le, Broger Tobias, Abubakar Ibrahim, Hayward Andrew, Thomas Bruce V, Temesgen Zelalem, Quraishi Subhi, von Delft Dalene, Jaramillo Ernesto, Weyer Karin, Raviglione Mario C (2016). Digital health for the End TB Strategy: developing priority products and making them work. Eur Respir J.

[46] Free C, Phillips Gemma, Watson Louise, Galli Leandro, Felix Lambert, Edwards Phil, Patel Vikram, Haines Andy (2013). The effectiveness of mobile-health technologies to improve health care service delivery processes: a systematic review and meta-analysis. PLoS Med.

[47] Mangan J, Burzynski J, deCastro B Rey, Salerno M M, Lam C K, Macaraig M, Reaves M, Kiskadden-Bechtel S, Bowers S, Sathi C, Dias M P, Goswami N D, Vernon A (2023). Challenges associated with electronic and in-person directly observed therapy during a randomized trial. Int J Tuberc Lung Dis.

[48] Vernon Andrew, Fielding Katherine, Savic Rada, Dodd Lori, Nahid Payam (2019). The importance of adherence in tuberculosis treatment clinical trials and its relevance in explanatory and pragmatic trials. PLoS Med.

[49] Kara G, Yalcin B. M. (2022). Comparison of in-person vs. video directly observed therapy (VDOT) on stigma levels in tuberculosis patients. J Am Board Fam Med.

[50] Lippincott Christopher K, Perry Allison, Munk Elizabeth, Maltas Gina, Shah Maunank (2022). Tuberculosis treatment adherence in the era of COVID-19. BMC Infect Dis.

[51] DiStefano M, Schmidt Harald (2016). mHealth for tuberculosis treatment adherence: a framework to guide ethical planning, implementation, and evaluation. Glob Health Sci Pract.

[52] Sekandi J, Murray Kenya, Berryman Corinne, Davis-Olwell Paula, Hurst Caroline, Kakaire Robert, Kiwanuka Noah, Whalen Christopher C, Mwaka Erisa Sabakaki (2022). Ethical, legal, and sociocultural issues in the use of mobile technologies and call detail records data for public health in the East African region: scoping review. Interact J Med Res.

[53] O'Brien N, Li E, Chaibva CN, Gomez Bravo R, Kovacevic L, Kwame Ayisi-Boateng N, Lounsbury O, Nwabufo NFF, Senkyire EK, Serafini A, Surafel Abay E, van de Vijver S, Wanjala M, Wangari M, Moosa S, Neves AL (2023). Strengths, weaknesses, opportunities, and threats analysis of the use of digital health technologies in primary health care in the Sub-Saharan African region: qualitative study. J Med Internet Res.

