# Supplementary figures and images for: Effectiveness of a Mobile Health Intervention (DOT Selfie) in Increasing Treatment Adherence Monitoring and Support for Patients With Tuberculosis in Uganda: Randomized Controlled Trial
Source: JMIR Mhealth Uhealth. 2025 Jan 16;13:e57991. doi: 10.2196/57991 (PMC11783032; doi:10.2196/57991)

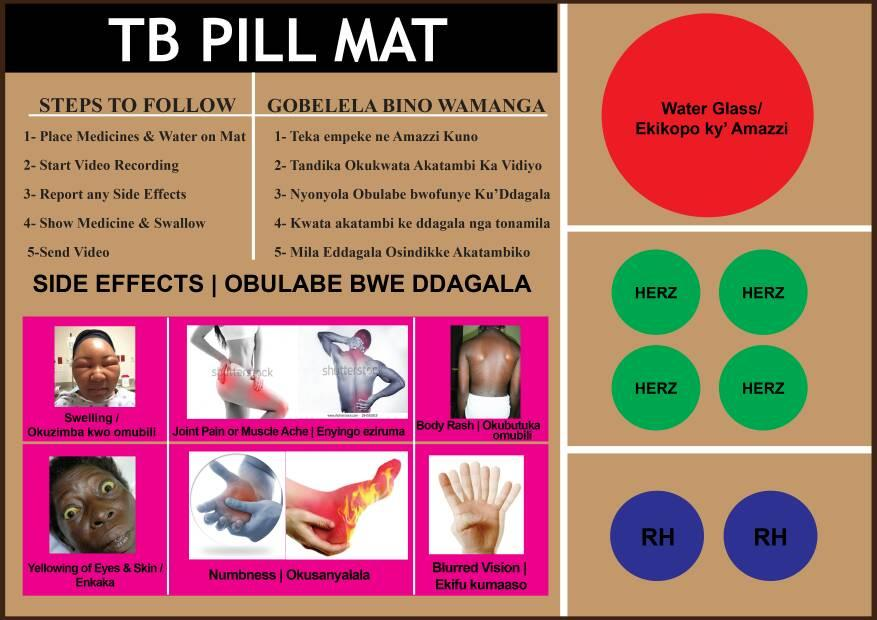

Supplement: Multimedia Appendix 2 [file mhealth_v13i1e57991_app2.png]
