# Supplementary material for: Effectiveness of a Mobile Health Intervention (DOT Selfie) in Increasing Treatment Adherence Monitoring and Support for Patients With Tuberculosis in Uganda: Randomized Controlled Trial
Source: JMIR Mhealth Uhealth. 2025 Jan 16;13:e57991. doi: 10.2196/57991 (PMC11783032; doi:10.2196/57991)
Supplement: Multimedia Appendix 3 [file mhealth_v13i1e57991_app3.pdf]

Protocol for Participant Follow-up of Missed Videos/ Follow up Visits

In case of missed videos or follow up visit, for VDOT the study staff will take the following actions:

1. Make two phone call attempts within the same day of an expected video, at one hourly intervals from last SMS text reminder at 2.00pm (3pm repeat at 4pm as needed).
2. After 24 hours (1 day of missed video), if no video is received two more phone calls at 3 hourly intervals should be made to follow up participants
3. After 48-hours (2 days of missed videos), if no video is received two more phone calls at 3 hourly intervals should be made to follow up participants including one call to the next of kin or treatment supporter.
4. After 72 hours (3 days of missed videos), then declare failure of phone contact follow-up of participant and then escalate to a field/home visit to reach the participant at their home or work.
5. The home or work visit should be conducted within the next 3-5 days to locate the participant and ascertain the medication adherence and reasons for missed videos.
6. If the participant is not located at home or work, 3 attempts can be made to locate the participants using the locator information, next of kin, treatment supporter, TB clinic and phone calls they provided with the next 5 days.
7. After 2 weeks of the intensive active follow-up protocol has been completed, there will be a waiting interval of 2 weeks with the hopes that the participant will call back or return to the clinic for a visit.
8. The TB clinic staff will be notified about the impending loss to follow-up and requested to send the patient to study staff once she/he returns for a prescription refill.
9. If a participant has not returned to the clinic for up to 2-month period since the last video/ clinic visit, he/she will be declared officially lost-to follow-up according to the standard definition by the TB program
10. However, the participant will not be terminated from the study until the end of his/her treatment period of 6 months or follow up period.
11. If the participant is located either by phone or field/home visit, the necessary support will be offered by study staff and documented in a follow-up log form A accordingly (See attached log form A).
12. A follow up log will be used to track follow up actions at the study site and in the field. The log is developed into an electronic format to be completed using a tablet as necessary.
